# Is long COVID the next global health crisis?

**DOI:** 10.7189/jogh.12.03067

**Published:** 2022-10-26

**Authors:** Mark A Faghy, Rebecca Owen, Callum Thomas, James Yates, Francesco V Ferraro, Lindsay Skipper, Sarah Barley-McMullen, Darren A Brown, Ross Arena, Ruth EM Ashton

**Affiliations:** 1 Biomedical Research Theme, School of Human Sciences, University of Derby, United Kingdom; 2 Healthy Living for Pandemic Event Protection (HL – PIVOT) Network, Chicago, Illinois, USA; 3 Patient and Public Representatives.; 4 Long COVID Physio, London, UK; 5 Therapies Department, Chelsea and Westminster Hospital NHS Foundation Trust, London, United Kingdom; 6 University of Illinois Chicago, Chicago, Illinois, USA

The rapid development and deployment of coronavirus disease 2019 (COVID-19) vaccines should be heralded as a feat of true scientific collaboration that saved millions of lives [1]. Despite the success, vaccines do not offer complete protection against infection, mild, and severe disease [2-5] and the development of a long-term complex symptom profile, commonly referred to as long COVID [5,6]. Long COVID results from a positive infection with severe acute respiratory syndrome coronavirus 2 (SARS-CoV-2), the virus that causes COVID-19. It used ribonucleic acid (RNA), which is prone to replication and proliferation, leading to the transmission of variants that are a threat to global health. SARS-CoV-2 is known to evolve at an approximate rate of 1.1 × 10 -3 substitutions per site per year, equivalent to a single substitution every 11 days [7]. Whilst not all mutations pose a threat to public and global health, previous variants including Omicron (B.1.1.529, BA.1, BA.1.1, BA.2, BA.3, BA.4, and BA.5 lineages) and Delta (B.1.617.2 and AY lineages) are widely regarded as variants of concern [8]. Data highlights that there is no difference in risk level between Delta and Omicron BA.1 variant among those that are triple-vaccinated [9]. These variants are characterised by several mutations that affect the spike protein and increase transmissibility [10], leading to the sustained and widespread transmission of acute infection which will inevitably result in increased progression of long COVID.

The latest data highlight that vaccines only offer a 15% reduction against developing long COVID and a 34% reduction in the risk of mortality [1]. Furthermore, clinical features are indistinguishable in those that were vaccinated and subsequently contracted SARS-CoV-2 compared with those with long COVID that were unvaccinated [5]. While data sets remain infrequent, >144 million people globally are living with multi-dimensional and episodic symptoms that broadly impact functional status and quality of life [11]. This is compounded by economic and societal drivers contributing to an increasing burden of Long COVID in the global population[12]. Data from Chen et al. [13] highlight that the global probability of developing long COVID is 0.43 (95% confidence interval (CI) = 0.39-0.46) with those hospitalised being more likely to develop lasting symptoms (0.54, 95% CI = 0.44-0.63) compared to those not hospitalised (0.34, 95% CI = 0.25-0.46). Symptom prevalence reported at 30 days post-infection is 0.37 (95% CI = 0.26-0.49), 0.25 at 60 days (95% CI = 0.15-0.38), 0.32 at 90 days (95% CI = 0.14-0.57) and 0.49 at 120 days 49 (95% CI = 0.40-0.59) [2]. Additional data highlights that the time to recovery exceeded 35 weeks in 3423 (91%) patients, reporting an average of 56 ± 26 symptoms across different organ systems [14]. The episodic nature of symptoms and functioning with long COVID is reported to affect 86% of participants (95% CI = 84.8%-87.0%) with symptoms triggered by exercise training, or physical or mental activity [14].

Easing COVID-19 restrictions is a complex, multifactorial, and often controversial decision; public health authorities, scientific advisory groups, and health care professionals expressed concern about relaxing restrictions, stressing a need for caution in the continued response to the pandemic [15]. The removal of social distancing measures, face mask mandates, and access to free testing in an attempt to restore pre-pandemic economic and social activities has resulted and will result in a sustained transmission of COVID-19, and subsequently, an increase in long COVID diagnosis. In the absence of pharmacological treatments or support mechanisms that address the disabling effects of long COVID, the burden upon public health and health care services in increasing [16]. There is a social and disability justice requirement to protect communities, especially vulnerable populations with magnified risks of post-COVID-19 infection complications [17]. Accordingly, we highlight critical areas of consideration informed by patient and public engagement representatives and two years of research into long COVID to 1) make the case that long COVID is arguably the next global health crisis, 2) raise the profile, awareness, and need for funding to support long COVID research; and 3) call for collaboration in the development of interdisciplinary approaches to address morbidity and disability in patients with long COVID.

## THE NEED FOR A UNIVERSAL DEFINITION FOR LONG COVID

In the initial stages of the pandemic, patients reported a broad reoccurring symptom profile that occurs in the weeks and months post-acute infection with COVID-19. Colloquially referred to as COVID long haulers, post-COVID condition/syndrome or long COVID, it is characterised by chronic, persistent, episodic, and disabling symptoms that have broad economic and health and well-being impacts [18]. In response to an emerging and novel condition, governments and global health agencies sought to produce a definition that encompassed the broad nature of long COVID. A lack of continuity is driven by a limited understanding of the condition and its impact on patients. Arguably the most detailed approach was undertaken by the World Health Organisation, who adopted a Delphi consensus methodology that incorporated five groups of stakeholders to produce a clinical case definition [19].

Post COVID-19 condition occurs in individuals with a history of probable or confirmed SARS-CoV-2 infection, usually 3 months from the onset of COVID-19 with symptoms that last for at least 2 months and cannot be explained by an alternative diagnosis. Common symptoms include fatigue, shortness of breath, and cognitive dysfunction but also others and have an impact on everyday functioning. Symptoms may be new onset following initial recovery from an acute COVID-19 episode or persist from the initial illness. Symptoms may also fluctuate or relapse over time.

World Health Organisation, (2021)

This is, to date, the most robust definition, as it encompasses the broad and changeable nature of the disease and the implications for patients, the authors also acknowledge that this is a temporary definition that will need to be revisited once more is known about the condition. As the knowledge base evolves, the definition must be refined to incorporate an increased understanding and must also be engrained with the patient’s voice and lived experience. There is a clear need for consistency in the development of a universal definition adoptable by governments and health care systems globally and used to support the service design and delivery of a growing global health challenge. While this might seem like a tall order, a consistent approach will ensure that long COVID patients have equitable access to the necessary treatments and support mechanisms needed to improve patient outcomes.

**Figure:**
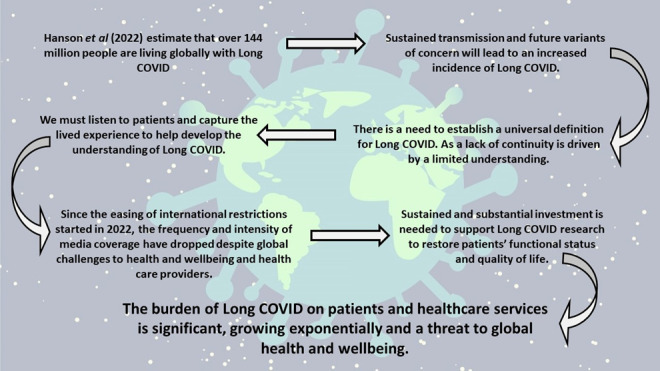
Photo: A summary of the key messages highlighted in the paper positioning Long COVID as a global health concern. Source: from the author’s own collection, used with permission.

## WE MUST INCORPORATE THE LIVED EXPERIENCE

The lived experience is defined as personal knowledge gained through direct, first-hand involvement in everyday events rather than through representations constructed by other people [20]. Acknowledging the novel challenges posed by COVID-19 and long COVID, the lived experience through the lens of patients is crucial in the design and delivery of research to understand the challenges and in the development of subsequent support mechanisms. Patient and public involvement and engagement will provide an additional dimension to much-needed research and remove discrepancies to ensure that research addresses the patient’s needs. Furthermore, research engrained in patient and public involvement and engagement provides an opportunity to embed the lived experience within research, enabling those living with chronic issues to identify the questions and issues that matter to them. The following passage is from a patient with long COVID participating in ongoing research into the determinants of recovery of long COVID:

Being part of research into Long COVID has been a game-changer on my long COVID journey. Knowing that I was able to actively engage in a process that could give so much hope to other patients, especially when I thought COVID had taken everything I had to give, was an awakening experience for me.

## THE NEED TO INCREASE AWARENESS OF LONG COVID

Since the easing of restrictions in the UK and globally, the frequency and intensity of media coverage has reduced, with little or no mention of the sustained challenges faced by patients or health care providers. The restoration of economic and social activities is a driving force behind these decisions while the widespread impact of long COVID has been overlooked. In this context, public engagement initiatives must be prioritised to intensify the action needed to address the increasing burden of long COVID and to increase the public knowledge and awareness of the remaining threat to global health. The following passage is from an online patient survey of people with long COVID about “living with COVID-19”:

I watch people behaving as though the virus has gone. They have no idea that my reality could become theirs. If this is ‘learning to live with Covid’ I would not wish it on anyone.

Not only does long COVID drastically impact the global burden of disease, but it impacts health care services, which are historically underfunded and under-resourced. Furthermore, recent reports indicate that >200 000 UK frontline health care workers are off from work due to COVID-19-related issues, adding to existing pressures on capacity and service delivery [21]. Simultaneously, the National Health Service is attempting to clear a backlog of over 6 million elective treatments [22].

## SUSTAINED SUPPORT IS NEEDED FOR LONG COVID RESEARCH

The National Institute of Health Research and UK Research and Innovation demonstrated an agile response to the COVID-19 pandemic by allocating research funding to address the immediate burden of COVID-19. While this was important for an immediate response, the long-term and unanswered challenges of long COVID should be addressed with a sustained allocation of research funding. Currently, the National Institute of Health is the only health research authority to make a public sustainable commitment to provide $US1.15 billion in funding over four years for research into the long-term health consequences of SARS-CoV-2. Sustained research support is essential to addressing the needs of millions living with and those yet to develop long COVID. At present, a lack of mechanistic and clinical understanding has hampered the global response to long COVID and is in part contributing to the rise in confirmed long COVID diagnosis. In the absence of an appropriate understanding, it is difficult to design and develop support mechanisms that address the needs of patients and restorespre-COVID-19 quality of life. Accordingly, insight and knowledge from pre-existing chronic conditions, eg, postural tachycardia syndrome (PoTS), and myalgic encephalomyelitis or chronic fatigue syndrome (ME/CFS) have been incorporated into support and management approaches [23]. While symptom presentation for long COVID appears to overlap with these disorders and can provide a helpful tool to manage post-COVID-19 symptoms, longer-term bespoke approaches must be developed for long COVID. Furthermore, research is needed that investigates its impacts across the lifespan of children, adults, and older adults who have been affected in different ways, but have access to similar support services. Therefore, it is unsurprising that the global response to long COVID is fragmented and uncoordinated. The broad, complex, and multi-system experiences of patients are hindered by a relapsing and episodic presentation which provides difficulty in ascertaining and addressing causal mechanisms which have resulted in patients being discharged or even dismissed by health care systems [18,19].

There is a clear need to understand organ pathology and the mechanistic processes that are widely reported by long COVID patients and to support the development of pharmacological interventions. There is a promise that anti-viral medications may address viral persistence [24] thought to contribute to the development of long COVID. Clinical trials assess their efficacy are ongoing [25]. Future widespread trials and longitudinal observations are also needed to determine the extent and efficacy of alleviating long COVID symptoms, which must also consider representation from ethnic minority groups that have been disproportionally affected by transmission, severe disease, and mortality [26]. Accordingly, bespoke support mechanisms are needed, but must be informed by research to address holistic deconditioning and to determine safe and effective approaches that can restore functional status and quality of life [27]. The following passage is provided from our established group of PPIE representatives:

Research into Long COVID **MUST** continue. Creating funding to enable research, planning environments to disseminate outcomes, and taking evidence seriously is the only way to avoid a new secondary COVID health crisis.

The response to the long COVID conundrum, which feels reminiscent of the challenge that faced Alan Turing at Bletchley Park during World War 2, needs to be developed with true interdisciplinary collaboration [17,28]. Multidisciplinary teams (MDTs) have been suggested as a key for the development and delivery of long COVID support services, but we suggest that the very nature of multidisciplinary working will result in limited patient benefit due to a lack of consideration and interaction between all parties that will effectively be working in silos rather than in unison with each other. We suggest that approaches engrained in interdisciplinary thinking, understanding of patient needs, and broad expertise based on collaboration are more likely to be effective in addressing the burden of long COVID [29]. There is also a need to incorporate the lived experience of patients, as early attempts to re-purpose existing clinical interventions have been rejected by patients and, in worst cases, been dangerous [5]. Gorna et al. [30] propose a bespoke process that consists of physical assessment by consultant physicians with medical speciality to identify organ or multisystem dysfunction which is then used to inform individualised and broad rehabilitation plans to address broader issues of recovery. Long COVID is an extremely individual condition, with no place for a one-model-fits-all approach. Emphasizing the complexity and scale of the public health challenge ahead, systems science and implementation science approaches could be important in identifying stakeholder roles and responsibilities in the subsequent design, delivery, and evaluation of long COVID support services [31].

While there is a clear need to understand and address the clinical burden of long COVID, research should also consider the broader impacts of long COVID and its adverse social and economic effect on children, families, and employment. The drive to restore pre-COVID-19 social and economic activity was part of the reason for removing all restrictions in the UK and globally. The long-term debilitating symptom profile that is widely reported will undoubtedly have a lasting impact on social and economic activities and must be considered in the long COVID global health crisis.

## CONCLUSION

The need to restore the functional status of long COVID patients is crucial for global and public health. Coupled with the removal of COVID-19 restrictions, sustained transmission, and future variants of concern, long COVID prevalence will increase affecting, hundreds of millions of people worldwide. Public recognition of disabilities associated with long COVID, alongside the urgent resources and collaborative research is required to develop treatments and support mechanisms that can better address the growing and future burden of long COVID.
